# Stacked ensemble machine learning for porosity and absolute permeability prediction of carbonate rock plugs

**DOI:** 10.1038/s41598-023-36096-2

**Published:** 2023-06-17

**Authors:** Ramanzani Kalule, Hamid Ait Abderrahmane, Waleed Alameri, Mohamed Sassi

**Affiliations:** 1 Department of Mechanical Engineering, Khalifa University, Abu Dhabi, UAE; 2 Department of Petroleum Engineering, Khalifa University, Abu Dhabi, UAE

**Keywords:** Core processes, Geology, Petrology

## Abstract

This study employs a stacked ensemble machine learning approach to predict carbonate rocks' porosity and absolute permeability with various pore-throat distributions and heterogeneity. Our dataset consists of 2D slices from 3D micro-CT images of four carbonate core samples. The stacking ensemble learning approach integrates predictions from several machine learning-based models into a single meta-learner model to accelerate the prediction and improve the model's generalizability. We used the randomized search algorithm to attain optimal hyperparameters for each model by scanning over a vast hyperparameter space. To extract features from the 2D image slices, we applied the watershed-scikit-image technique. We showed that the stacked model algorithm effectively predicts the rock's porosity and absolute permeability.

## Introduction

Determining geological rock properties such as absolute permeability and rock porosity is essential for oil and gas reservoir production, enhanced oil recovery, and CO_2_ injection and hydrogen storage^1–3^. Estimating reservoir properties can be challenging due to the heterogeneities and complexity of the reservoir rock structures, which can vary significantly across different geological formations and burial histories^4^. Rock properties such as permeability can be determined experimentally in the laboratory by conducting core flooding. However, experiments are time-consuming, labour-intensive, and expensive^5,6^. Rock properties can also be estimated using numerical simulations; however, these methods require extensive computational resources and numerical skills to set up the simulations^7,8^. Recently, Digital rock physics (DRP) has been established as an efficient workflow to estimate the petrophysical properties of the rock sample, particularly in the case of homogenous rocks^9,10^. DRP relies on advanced imaging approaches, image processing techniques, and computational methods. The high-resolution digital images of pores and grains structures are used to conduct numerical simulations at the pore scale and infer rock properties such as porosity and directional permeability^11–22^. However, in the case of heterogeneous carbonate rocks comprising micro and nano-pores, predicting the rock properties using DRP workflow can have significant uncertainties^4,13^.

Several empirical and theoretical models correlate porosity, permeability, and other reservoir-based properties^23–25^. However, the generalizability of these correlations is limited because several reservoir property relationships are complex and nonlinear. Therefore, properties such as permeability cannot accurately be estimated using simplified or linear relationships. Machine learning (ML) and deep learning (DL) approaches are considered alternatives to overcome the nonlinear dependencies of the properties of the rock structure. ML approaches aim to extract statistical patterns from CT images and correlate them to the rock properties. The efficiency of the ML model depends on its generalizability, i.e., making accurate predictions based on unseen structures and features.

ML and DL approaches can predict multiple rock properties from various rock samples in a few seconds with limited computational resources^2,26–30^. This presents a significant advantage compared to experimental measurements and numerical simulations, which do not allow the characterization of more than one reservoir sample at a time. Several successful studies on predicting porosity and absolute permeability from rock images using ML are found in the literature. For instance, Araya-polo et al.^31^ used DL to predict absolute permeability from 2D high-resolution images. They showed that DL accurately predicts absolute permeability in seconds. Wu et al.^32^ proposed a physics-informed Convolutional neural network (PIML-CNN) algorithm to improve the accuracy of the conventional convolutional neural network (CNN) algorithm in predicting absolute permeability. They showed that DL efficiently estimates absolute permeability compared to flow dynamics simulations and the Kozeny-Carman equation. Alqahtani et al.^33^ used CNNs to estimate porosity using 2D image slices of Berea Sandstone with or without image segmentation^34^. Their results portrayed a good agreement with ground truth labels. Similarly, Alqahtani et al.^33^ applied CNNs to 2D greyscale micro-CT rock images^33^. They predicted porosity with a less average error compared to the experimental measurements. Finally, Tembely and Alsumaiti^35^ applied shallow learning and DL algorithms to 3D micro-CT images to determine absolute rock permeability. They observed that shallow learning combined with gradient boosting (GB) performs well concerning their predictions of absolute permeability. Additionally, they observed better performance from deep neural networks (DNN) than gradient boosting with linear regression analysis.

Despite achieving impressive success, machine learning models often struggle with generalizability to new, unseen data due to overfitting and limited training datasets. These models can also be prone to biases and variances, negatively impacting their predictive accuracy. Ensemble learning has been proposed to minimize model variances and overfitting and provide better predictions^36–38^. Boosting, bagging, and stacking are some types of ensemble learning proposed in the literature. Stacking presents strong prediction capability because it integrates several model predictions into a single meta-leaner^39^. This approach improves the model generalizability and prediction accuracy of the meta-learner. Several studies have demonstrated the power of model stacking and other ensemble learning techniques in predicting different properties better than individual models^39–44^. Jian et al. (2020) studied the integration of DNNs and several ensemble learning machines in bagging and boosting types to estimate missing well logs. Results showed that combining several machine learning models can improve predictions. The application of the stacking method to predict petrophysical properties is very limited. Only one relevant study used stacking to estimate absolute permeability in heterogenous oil and gas reservoirs from well-log data^45^. The authors showed that their Ensemble model outperforms the individual models in terms of generalizability.

In this work, we leverage the advantages of the stacking approach, an ensemble learning algorithm, to predict absolute permeability and porosity from carbonate rock pore-scale features. We adopt six ML-based linear and nonlinear regression algorithms, including deep neural networks. We use averaged pore properties extracted from 2D slices of 3D micro-CT carbonate rock images using the watershed-sci-kit-image technique as input features to our proposed models. The rest of the paper is organized as follows. First, the methodology section highlights the methods and resources used in this work. Finally, the predicted results are presented and discussed in the third section.

## Methodology

**Figure 1:**
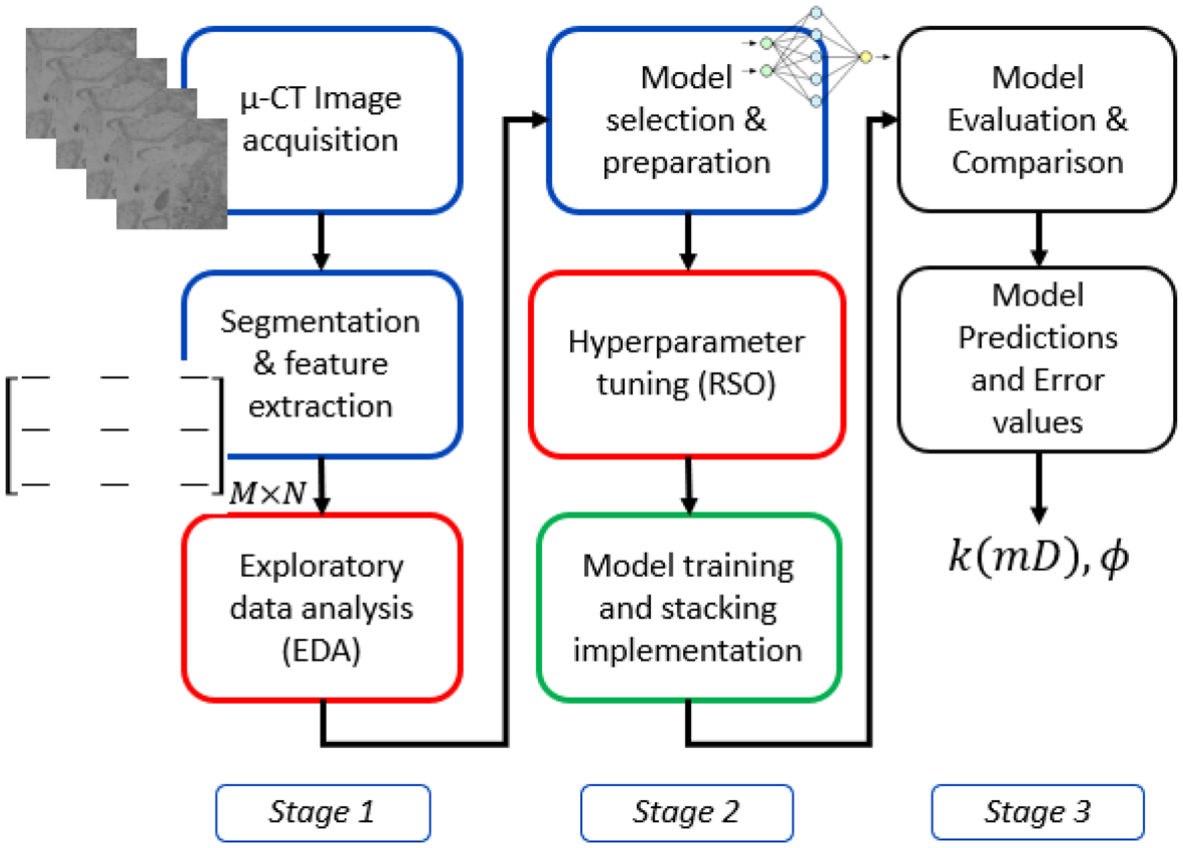
Proposed general flow chart.

This section discusses the approach and methodologies to predict rock porosity and absolute permeability. We first discuss the geological analysis of the core samples selected for the proposed dataset. Next, we present the laboratory methods for measuring rock porosity and absolute permeability. Finally, we present the image processing protocol, feature extraction methods, and used regression techniques. Figure 1 illustrates the proposed general flow chart of the study.

### Geological analysis of the dataset

**Figure 2:**
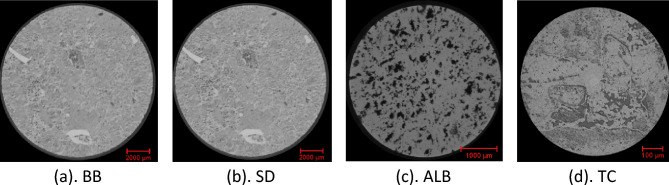
2D Micro-CT image slices of the selected carbonate rock samples at different imaging resolutions. (**a**) BB: 14.01 µm, (**b**) SD: 5.32 µm, (**c**) ALB: 0.81 µm, (**d**) TC: 3.93 µm.

**Figure 3:**
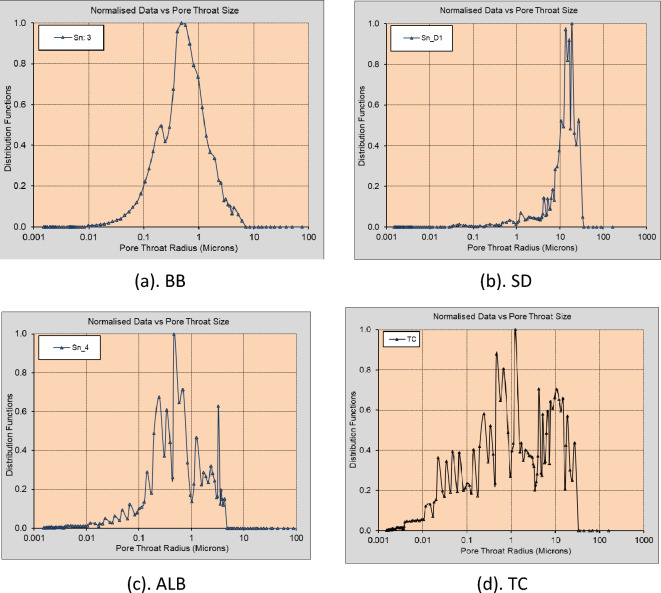
Pore-throat distribution plots of the selected rock samples.

Figure 2 shows typical 2D micro-CT image slices from the 3D CT scans of four core plugs selected for this study, namely, Silurian dolomite (SD), Albion-4 carbonate (ALB), and real middle eastern carbonate rocks (TC & BB). The rock samples, measuring $3.8\times 7.6\mathrm{ cm}$, were scanned at various resolutions using the Xradia Versa 500 Micro-CT machine to obtain high-resolution 3D scans. Each 3D image obtained from the micro-CT reconstruction procedure contains information about the local density of the rock sample, which can be visualized as a stack of 2D images^46^. These core samples were selected because they present different pore-throat distributions, various levels of heterogeneity, and a large range of permeability (10–400 mD). Figure 3a indicates the BB sample's pore size distribution, which ranges from 0.001 to about 0.9 µm. The pore size distribution of the SD sample ranges from 0.01 to 50 µm; see Fig. 3b. ALB sample displays a bimodal pore distribution around 0.01 µm and 8 µm, respectively; see Fig. 3c. The TC sample has a broad pore size distribution that ranges from 0.005 to 50 µm, see Fig. 3d, exhibiting higher levels of heterogeneity.

### Laboratory measurements

The porosity and absolute permeability of the four different heterogeneous carbonate rock samples were measured in the laboratory, and their values are summarized in Table 1. Based on Boyle's law, the rock porosity was determined using a helium porosimeter. Mercury Injection Capillary Pressure (MICP) tests were conducted on the trimmed samples from the four rock samples. The MICP porosity obtained corresponds to the effective porosity and does not include uninvaded or isolated pores. The absolute permeability is estimated using water (brine) injection pressure drop results at different flow rates and Darcy's law.

**Table 1:** Experimental values for the selected samples.

Sample	Resolutions (µm)	Experimental values	
		Porosity	Permeability (mD)
BB	14.01, 3.92	0.257	11.30
SD	13.24, 5.32	0.158	278.85
ALB	13.44, 4.24, 0.81	0.208	10.23
TC	3.93, 0.94	0.256	336.94

### Image processing

**Figure 4:**
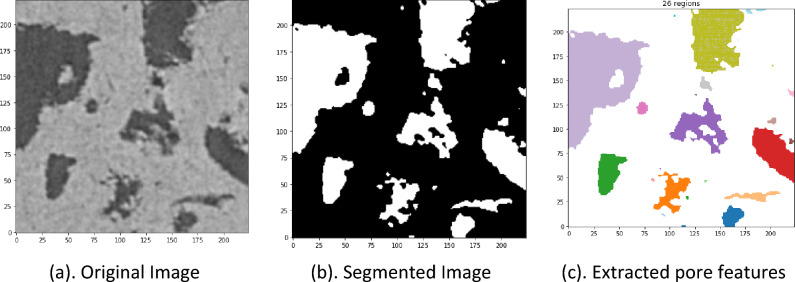
Original image, segmented image, and extracted regional (pore) features or properties.

The image processing techniques include image denoising, removal of artifacts, and classifying pixels into representative clusters^34^. These consist of converting images into pores and rock matrices. Image processing techniques are either manual or automatic^19^. The manual segmentation algorithms are usually subjective and depend on the operator's experience. Moreover, the manual segmentation algorithms cannot be generalized to all samples^33^. On the other hand, automatic segmentation algorithms are less subjective, more efficient, and generalizable^47,48^. As a result, automated segmentation algorithms are more implemented in the DRP workflow^49^. In this study, we apply the Otsu localized algorithm, an efficient automatic segmentation algorithm, to the watershed image segmentation technique to segment the selected images. This segmentation approach is less subjective to the operator's inputs than several conventional methods^6^. Furthermore, the proposed method can reduce binarized image noise and retain much of the original image information^50^. Figure 4b presents an example of a segmented image obtained from the original image in Fig. 4a using the proposed algorithm.

### Feature extraction using watershed scikit-image technique

The watershed technique extracts the regional features (RegionProps) of image pores from each 2D image as a dimensional parameter. The watershed function is implemented in the scikit-image Python module. This function allows the calculation of useful dimensional parameters, including area, equivalent diameter, orientation, major axis length, minor axis length, and perimeter, among others, that are evaluated for the different pores in each image. Here, fourteen RegionProps features were extracted. These features represent compact and informative descriptions of the objects in the image and are used to reduce a high-dimensional micro-CT image into a lower-dimensional feature space to ease the analysis. The average proportions of these different regional parameters from each image are evaluated and stored in a matrix (6500 X 14); the number of images in the dataset by the fourteen features columns. Figure 4a shows an example of a 2D 224X224 slice of an original image. Figure 4 represents a watershed segmented image, while Fig. 4c presents a visual of the various extracted pores from the segmented image.

### Exploratory data analysis (EDA)

We conducted an EDA on the extracted features in which a feature correlation analysis is performed to reduce the number of features into a subset of strongly correlated features to the target. To understand the relationships between input features and minimize multicollinearity, we performed hypothesis testing with statistical inference analysis at a 0.05 level of significance (*p*-value). This selection of the significance level is entirely based on literature as a commonly used threshold in hypothesis testing^51,52^. We adopt the weighted squares statistical regression model^53^ to identify the most relevant features to the target features. Moreover, we implemented the Variance Inflation Factor (VIF) to minimize multicollinearity between features.

### Stacked generalization

Stacking (stacked generalization) is an ensemble machine-learning algorithm that blends various estimator predictions in a meta-learning algorithm. This technique combines predictions of heterogenous weaker learners in parallel as features and outputs for a better singular (blender or meta-learning model) prediction^42^. Combining these different models with different strengths and weaknesses can give a better prediction with minimal variances than a single model, mitigating overfitting, improving model robustness, and minimizing misleadingly high model performance scores^42^. This approach involves two levels. Level 1 involves several ML and/ or DL models trained independently on the same dataset for a unique performance score. Level 2 consists of a meta-learner that leverages the individual performances of the previously trained models in level 1 and trains on the same dataset to provide an improved performance score^41^.

**Figure 5:**
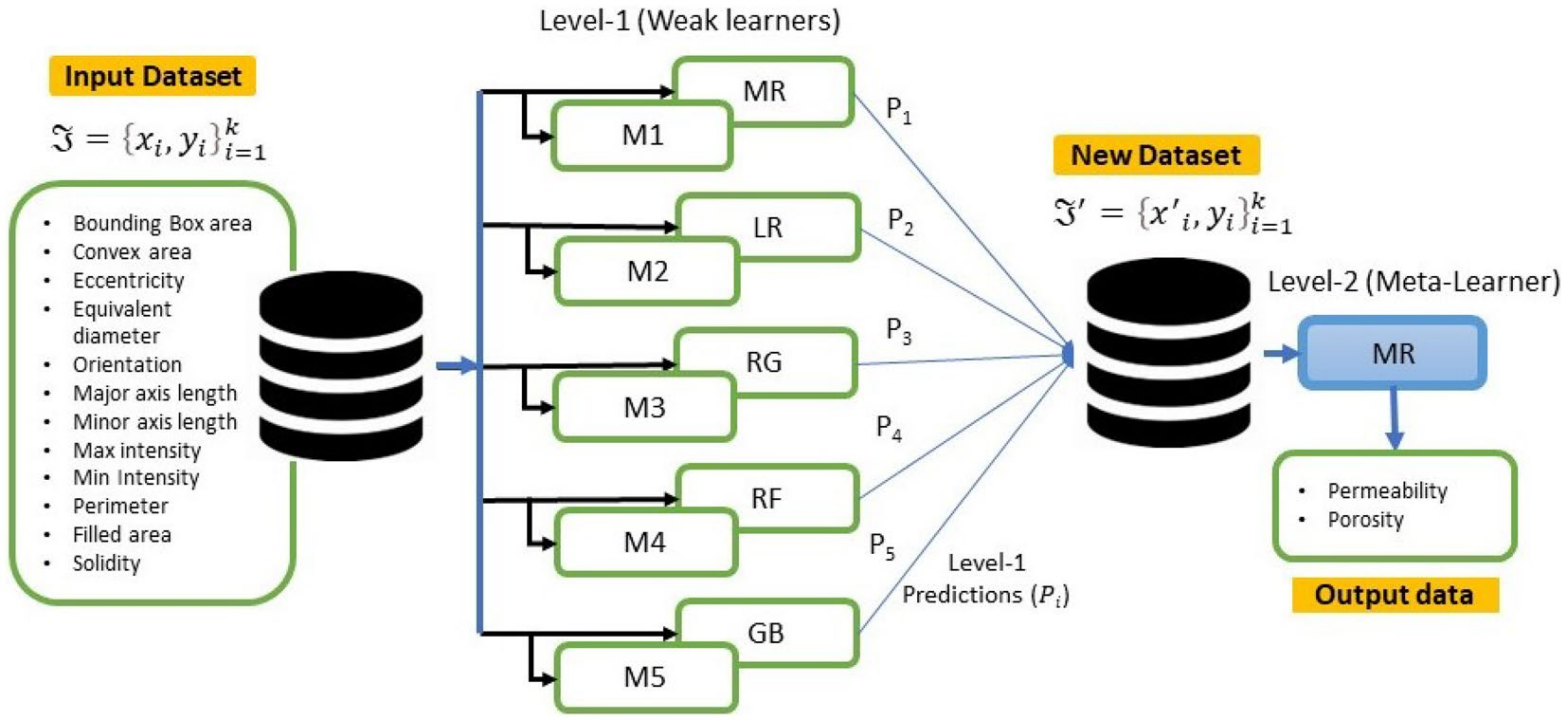
A stacked generalization illustration.

A summarized stacking regression approach is presented in Table 2 and illustrated in Fig. 5. Considering cross-validation over the training dataset, the original dataset will be sliced into k-folds or partitions $\Im = (\Im_{1} ,\Im_{2} , \ldots ,\Im_{k} )$. Therefore, when trained on a given dataset $\Im_{i}$ and tested on, $\Im_{ - i}$ the first weak learner $M_{1}$ will provide an output $M_{1} (x_{i} )$. In this case, the new dataset $\Im^{\prime} = \left\{ {x^{\prime}_{i} ,y_{i} } \right\}_{i = 1}^{k}$ $\mathop{\longrightarrow}\limits^{{}}(x_{i} \in \Re^{n} ,y_{i} \in \Re^{n} )$ will be generated from predictions of weak learners $M_{n}$, as in Table 2.

**Table 2:**
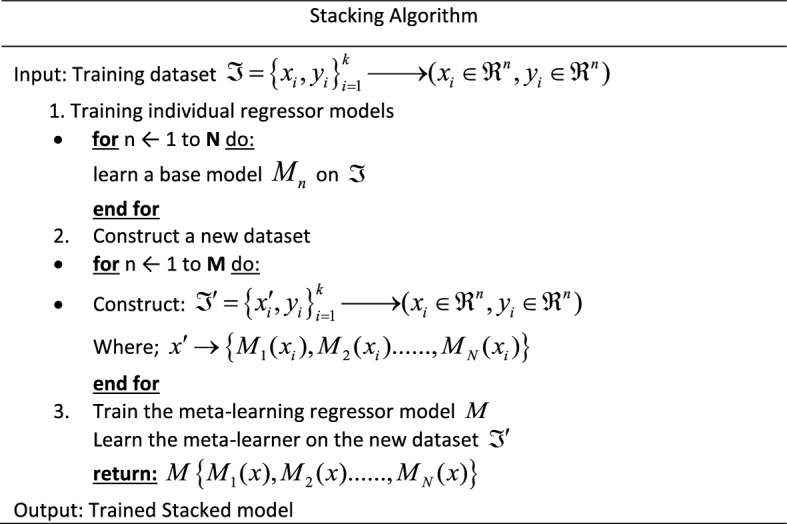
A summarized stacking generalization approach.

In the literature, it is common practice to have a heterogeneous combination of base (weaker learners) models^36^. However, this is not the only option since the same type of model, such as the DNN, can be used with different configurations and trained on different parts of the dataset. Therefore, we used both practices in this study to evaluate their influence on model accuracy, predictions, and computational requirements. Below we present the capabilities of six (6) ML regression models adopted for stacking and predicting permeability and porosity. The machine models adopted include linear and nonlinear regression models discussed below.

- 1. Multiple linear regression (MR) is the most basic ML model with a single predictor variable that varies linearly with more than one independent variable. It assumes little or no multicollinearity between the variables, and the model residuals must be normally distributed. The main objective is to estimate the intercept and slope parameters defining the straight line best fitting the data. The most common method used to calculate these parameters is the least squares method, which minimizes the sum of the squared errors between the predicted and actual values of the dependent variable. The objective function is given in Eq. 1, with the λ (tuning parameter) set to zero.
- 2. Ridge regression (RG) is an enhancement to MR, where the cost function is altered by incorporating a penalty term (L2 regularization) which introduces small amounts of bias to reduce the model complexity and improve predictions. If λ (tuning parameter or penalty) is set to zero in Eq. 1, the cost function equation reduces to the MR model. Here, ${\mathrm{x}}_{\mathrm{ij}}$ are the m explanatory variables, $\mathrm{e}$ is the error value between the actual and predicted, while ${\mathrm{y}}_{\mathrm{i}}$ is a dependent variable. ${\mathrm{b}}_{\mathrm{j}}$ represents a set of model parameters to be estimated to minimize the error value. The cost function is expressed as.
  1 $$\sum\limits_{i = 1}^{n} {\left( {y_{i} - \hat{y}_{i} } \right)^{2} } = \sum\limits_{i = 1}^{n} {e^{2} } = \sum\limits_{i = 1}^{n} {\left( {y_{i} - \sum\limits_{j = 0}^{m} {x_{ij} b_{j} } } \right)^{2} } + \lambda \sum\limits_{j = 0}^{m} {b_{j}^{2} }$$
- 3. Lasso regression (LR): (Least Absolute and Selection Operator) is another regularized approach of MR. Unlike RG, which involves a penalty to reduce model complexity and avoid overfitting, LR considers the absolute form of the individual feature weights (see Eq. 2). The cost function of LR is expressed as:
  2 $$\sum\limits_{{i = 1}}^{n} {\left( {y_{i} - \hat{y}_{i} } \right)^{2} } = \sum\limits_{{i = 1}}^{n} {\left( {y_{i} - \sum\limits_{{j = 0}}^{m} {x_{{ij}} b_{j} } } \right)^{2} } + \lambda \sum\limits_{{j = 0}}^{m} {\left| {b_{j} } \right|}$$
- 4. Random Forest Regression (RF): The RF is the most widely used machine learning algorithm because of its simplicity and high accuracy on discrete datasets; it is also computationally cheaper to apply. **RF** technique is employed to decorrelate the base learners by learning trees based on a randomly chosen subset of input variables and a randomly chosen subset of data samples^54^. The algorithm for training a greedy decision tree is presented in Table 3. The RF algorithm follows two essential aspects: the number of decision trees (estimators) required and the average prediction across all estimators. The ensembled estimators can introduce randomness to the model while mitigating overfitting and improving model accuracy.
- 5. Gradient Boosting Regression (GB): The GB Algorithm (Table 3) is a machine learning algorithm for classification and regression problems. In Gradient Boosting Regression, a sequence of weak decision tree models is created in a step-by-step fashion, where each model attempts to correct the errors made by the previous model. First, this technique is trained on a continuous dataset to provide given output/s by an ensemble of several weaker learners (boosting), such as decision trees, into a stronger learner. Then, at a constant learning rate, the weak learners are fitted to predict a negative gradient updated at every iteration by a loss function. This algorithm is widely used due to its computational speeds and interpretability of the prediction^55^.

**Table 3:**
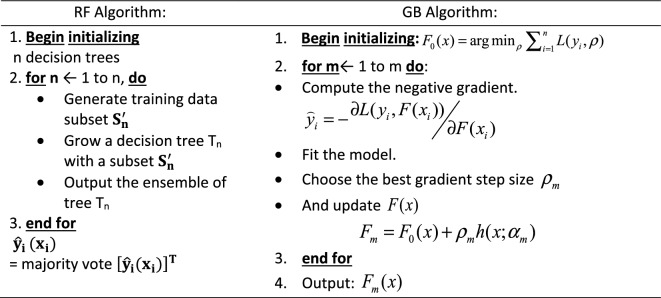
RF and GB algorithmic definitions.

**Figure 6:**
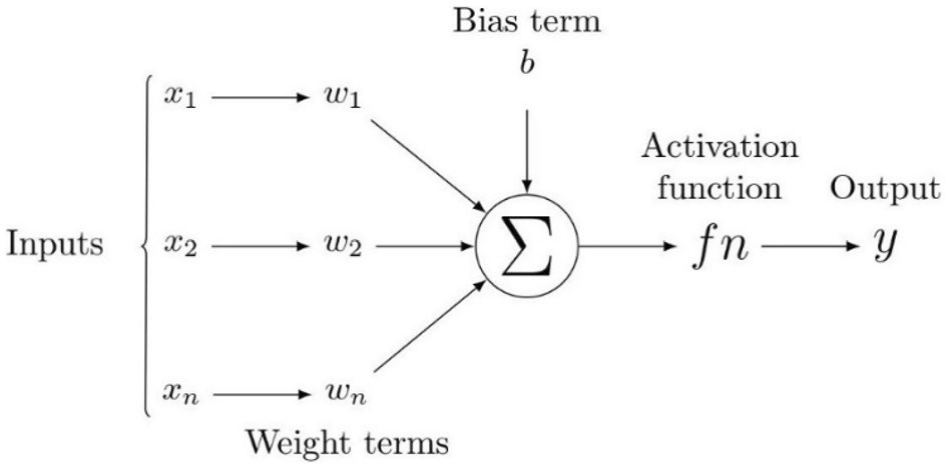
A schematic diagram of a neural network.

DNNs have been recognized as powerful tools that provide accurate predictions in classification and regression problems in several scientific fields. For example, DNNs have been applied in petroleum engineering to predict different reservoir rock properties from well-logging resistivity measurements, seismic data, and numerical or experimental measurements^56^. Figure 6 presents an illustration defining the flow chart of neural networks. Here, all inputs are multiplied with their corresponding weights representing the strength of neurons and are controlled by a cost function. A weighted sum then adds together the multiplied values. The weighted sum is then applied to an activation function that delivers the network's output. Considering a DNN with multiple output targets, the corresponding cost function based on mean square training errors is given as:

3 $$J(\theta ) = \frac{1}{2}\sum\nolimits_{d \in D} {\sum\limits_{i = 1}^{k} {\left( {\overset{\lower0.5em\hbox{$\smash{\scriptscriptstyle\frown}$}}{y}_{id} - y_{id} } \right)^{2} } }$$

where $$\overset{\lower0.5em\hbox{$\smash{\scriptscriptstyle\frown}$}}{y}_{id}$$ are the target values, and $$y_{id}$$ are the network outputs associated with the network output $$k$$ and training example $$d$$. The gradient descent rule is used to find hypothesis values to the weights that will minimize $$J(\theta )$$. Table 4 shows the backpropagation algorithm used to find these weights. The weight-update loop in backpropagation may be iterated thousands of times in a typical application. A variety of termination conditions can be used to halt the procedure.

**Table 4:**
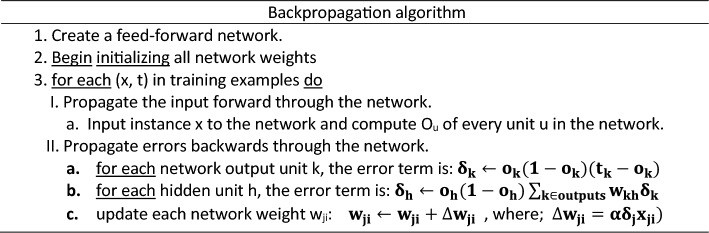
The backpropagation algorithm of neural networks.

The study also adopts DNNs as a regression approach to map the extracted features to absolute permeability and porosity. We train optimum DNN models (M_1_–M_5_) of a different number of hidden layers and the number of perceptrons in each layer to affect the model performance score. During the training of each model, we investigated and adopted the optimum hyperparameters of batch size, number of epochs, and a suitable optimizer for each model through a constrained randomized search (RSO) approach.

The ensemble stacking approach is designed to stack multiple predictions from three (3) linear and two (2) nonlinear machine learning-based models into a meta-leaner linear model (SMR-ML). The method is also designed to stack various predictions from multiple DNN networks of various levels of model complexity (the number of hidden layers and perceptrons per layer (Table 5). Individual predictions (${P}_{1}-{P}_{5}$) from the five DNN model (${M}_{1}-{M}_{5}$) architectures are stacked together into a meta-leaner linear model (SMR-NN). Each model is trained and saved independently on an optimum hyperparameter space in both stacking cases. To demonstrate the capabilities of the proposed approach, we select the multiple linear regression model (SMR) as the meta-learning model^57^.

**Table 5:** DNN model architectures.

Model (M)	Hidden layer 1	Dropout layer 1	Hidden layer 2	Dropout layer 2
1	128	–	–	–
2	128	–	64	–
3	224	–	128	–
4	128	0.1	64	0.1
5	224	0.2	128	0.2

### Hyperparameter tuning

Hyperparameters, such as the size of the network, the learning rate, the number of layers, and the type of activation function, control the learning process of a machine learning model. By adjusting these parameters, the model's performance can be improved. Hyperparameter tuning, the process of identifying the best training hyperparameters of a single model, is tedious and usually based on trial and error. However, it is possible to recommend searching the hyperparameter space for the best hyperparameters that can deliver the best model score. Two generic tuning methods widely used include the exhaustive grid search (EGS) and the randomized parameter optimization (RSO). The EGS is a compelling approach but computationally expensive^58,59^. In this study, we adopt the randomized parameter optimization method, which implements a randomized parameter search over selected model hyperparameters. Compared to the EGS, the addition of none influencing parameters into the pool of RSO-selected parameters does not affect the efficiency of the approach. Note that the selected best hyperparameters are entirely based on the dataset used and may change for other datasets.

### Metrics and hyperparameters

This study adopts the mean squared error (MSE) as a loss function. MSE is widely used in ML-based regression models. The MSE gives the mean value of the square differences between the target set points and the regression line, expressed in Eq. (4).

4 $$\theta = \arg \min _{{w,b}} \frac{1}{N}\sum\limits_{{i = 1}}^{N} {\left( {l_{i} - p(x_{i} ,\theta )} \right)^{2} }$$

Additionally, we adopt the mean absolute error (MAE) function (Eq. 5), a metric related to the mean of the absolute values of each prediction error on the test data. P is the property operator, which is a function of the inputs and the weights of the predictor network. This may also be identified as an activation function. Θ denotes the model weights, l_i_ represents the actual labels, and N represents the dataset size.

5 $$\theta = \arg \min_{w,b} \frac{1}{N}\sum\limits_{i = 1}^{N} {\left| {\left( {l_{i} - p(x_{i} ,\theta )} \right)} \right|}$$

Typically, when conducting regression analysis with multiple inputs, it is advisable to rescale the input dataset to account for variations in their influence on the dependent variable^60^. We tested various scaling techniques, including min–max scaling, absolute maximum scaling, and standardization. Based on our evaluation, standardization, which transforms the data to a normal distribution, yields the best results. Hence, we applied standardization (Eq. 6) to the dataset before training and evaluating the regression models discussed^61^. A dataset split of 80:20 in percentage is considered for the training and testing of the models. In Eq. (6), x represents the model inputs, µ denotes the mean, and σ is the standard deviation of the data.

6 $$x_{i - scaled} \xleftarrow{new}\left[ {\frac{{x_{i} - \mu }}{\sigma }} \right]_{stdn}$$

The proposed models are trained and evaluated against test data using the coefficient of determination (R^2^) see Eq. (7). R^2^ is a goodness-of-fit measure of the model predictions to the actual targets. It ranges between 0 and 1 or is expressed as a percentage. The higher the R^2^, the more accurate the model is in predicting the targets, where $$y_{i} ,\overset{\lower0.5em\hbox{$\smash{\scriptscriptstyle\frown}$}}{y}_{i}$$ and $$\overline{y}_{i}$$ represent the targets, predictions, and mean values, respectively.

7 $$R^{2} = 1 - \frac{{\sum\nolimits_{i} {\left[ {y_{i} - \overset{\lower0.5em\hbox{$\smash{\scriptscriptstyle\frown}$}}{y}_{i} } \right]}^{2} }}{{\sum\nolimits_{i} {\left[ {y_{i} - \overline{y}_{i} } \right]}^{2} }}$$

The proposed models are implemented using the Python platform. The RSO hyperparameter search is done using a single CPU node of a high-performance computer (HPC). Model training and testing were done using a NVidia GeForce Titan graphics card system with 12 Gigabyte memory, core i7 of 8th generation.

## Results

Several ML models, including DNNs, have been optimally trained on the dataset of extracted features (pore properties). These features were extracted from 2D slices of 3D micro-CT images from four carbonate rock samples. The selected carbonate rock samples were scanned at various image resolutions, representing a wide range of pore throat distributions and different levels of heterogeneity. Finally, the trained models are tested on unseen 2D slices to predict both porosity and absolute permeability for single and multi-output considerations.

**Figure 7:**
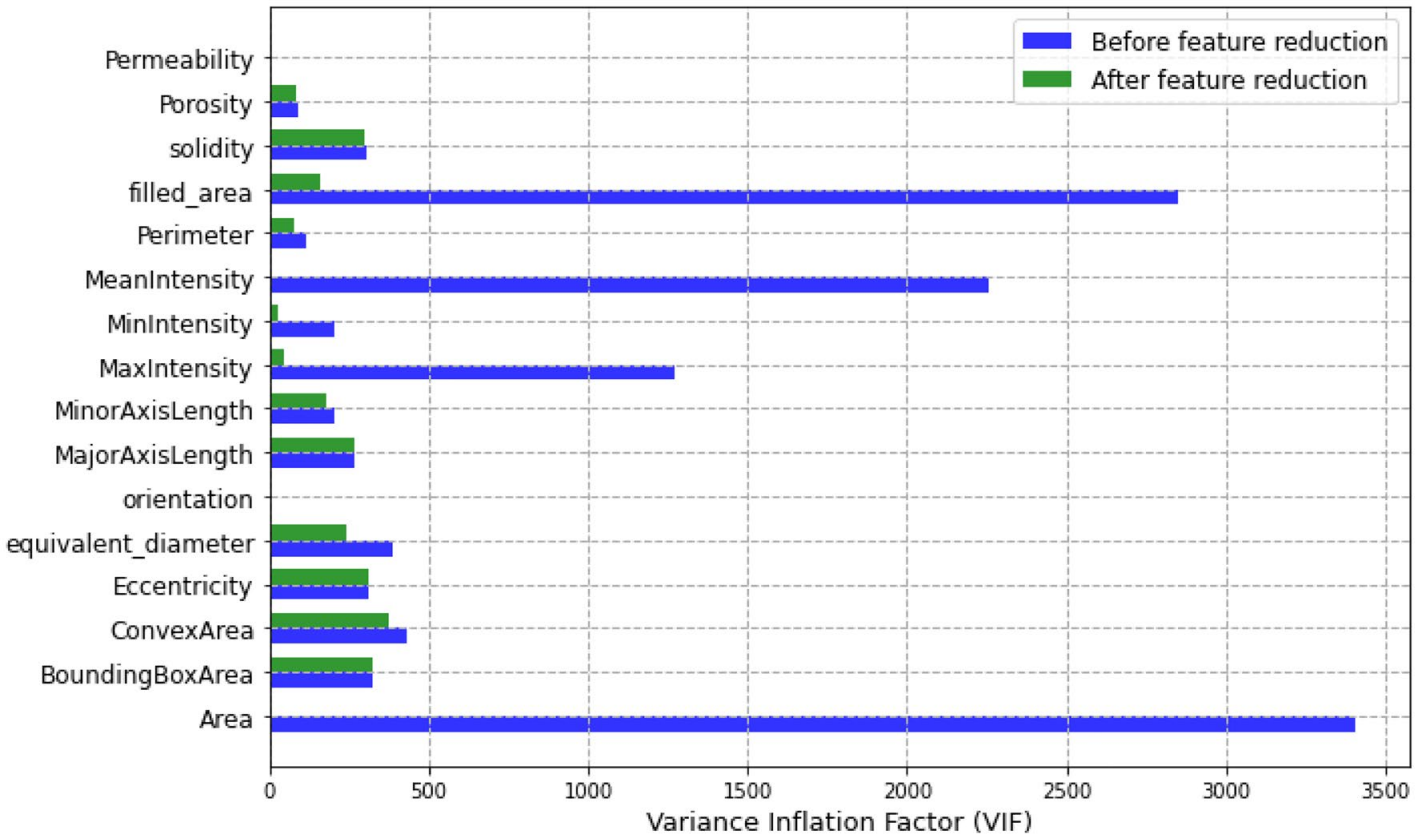
Variance inflation factor (VIF) before and after feature reduction.

In the EDA, we identified the most important features that were highly correlated with permeability and porosity. However, we also noticed that some features exhibited high multicollinearity between them, leading to unstable model predictions and inflated errors. To mitigate this issue, we dropped some of the highly correlated features, such as the area and the mean Intensity, while also considering the relevance of all features to the target predictions. By doing so, we could select a set of features that maximized the predictive power of the models while minimizing multicollinearity. The remaining features included the bounding box area, the convex area, eccentricity, equivalent diameter, orientation, perimeter, filled area, solidity, major and minor axis length, minimum and maximum Intensity. Figure 7 presents the differences between the VIF before and after feature reductions. The plot shows that there was a significant reduction in VIF values after dropping the highly correlated features.

For each of the selected models, we identify an optimal hyperparameter space from a vast array of significant hyperparameters for each proposed model. We used five-fold cross-validation RSO over a good grid of parameter values and functions and hundred iterations. As a result, the model generates a unique set of optimal hyperparameters for every iteration with a particular fold. This enhances precision, performance, and shorter training periods. Table 6 presents a set of evaluated optimal hyperparameters for each model selected in SMR-ML. Regarding the SMR-NN, by fixing the model architectural structures (Table 5), we identify a set of optimal hyperparameters for each of the selected DNN models based on the dataset. Table 7 presents the results obtained from the selected hyperparameter space and the best score (R^2^) based on two outputs of both porosity and permeability.

**Table 6:** ML: model optimal hyperparameters.

Model	Hyperparameter space
MR	normalize = True, fit intercept = True
LR	n_alphas = 50, max_iter = 2000, eps = 0.0001, cv = 3
RG	normalize = True, fit intercept = True, cv = 3
RF	n_estimators = 1400, min_samples_split = 2, min_samples_leaf = 1, max_features = 'auto', max_depth = 100, bootstrap = True
GB	n_estimators = 1200, min_samples_split = 5, min_samples_leaf = 4, max_depth = 10, loss = 'ls', learning_rate = 0.1, criterion = 'mse'
SMR-ML	normalize = True, fit intercept = True

**Table 7:** SMR-NN: DNN optimum hyperparameters.

Model	Optimizer	Epochs	Batch size	Best Score (R^2^)
M 1	Adam	150	64	0.77
M 2	Adam	150	32	0.96
M 3	Adamax	150	32	0.97
M 4	Nadam	150	32	0.96
M 5	Adam	100	128	0.96
SMR-NN	normalize = True, fit_intercept = True			0.96

**Figure 8:**
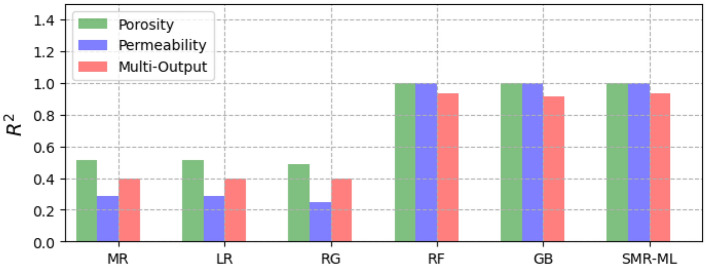
SMR-ML model performances with different target configurations.

**Figure 9:**
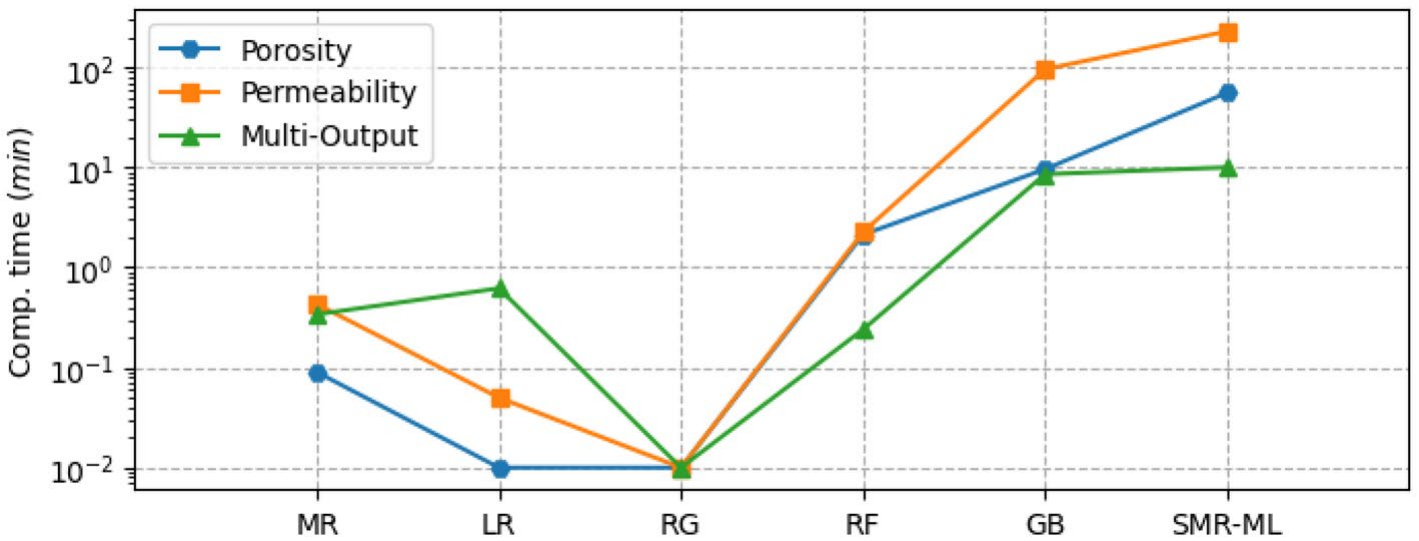
SMR-ML computational time requirements with different target configurations.

**Figure 10:**
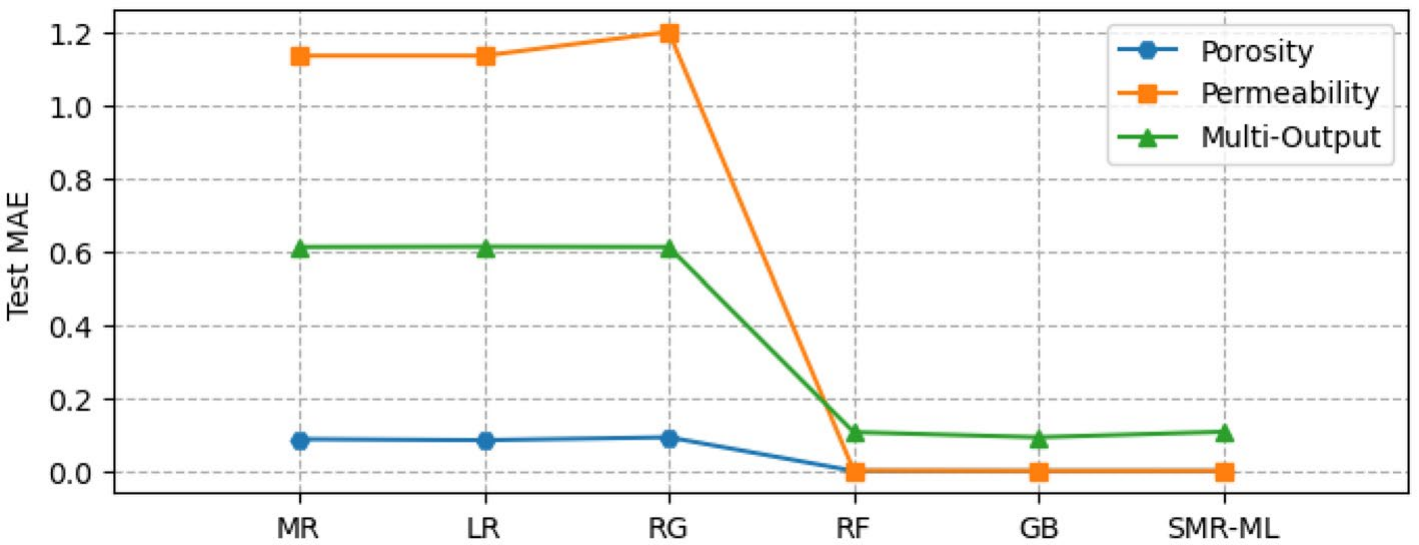
SMR-ML test MAE with different target configurations.

**Figure 11:**
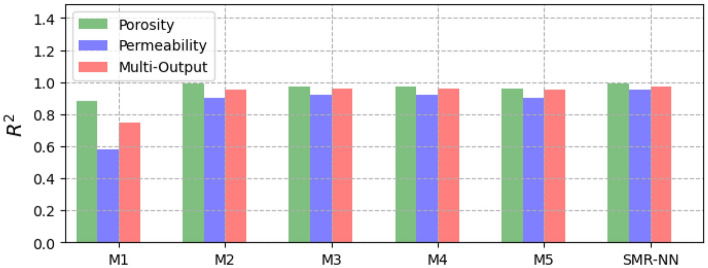
SMR-NN model performances with different target configurations.

**Figure 12:**
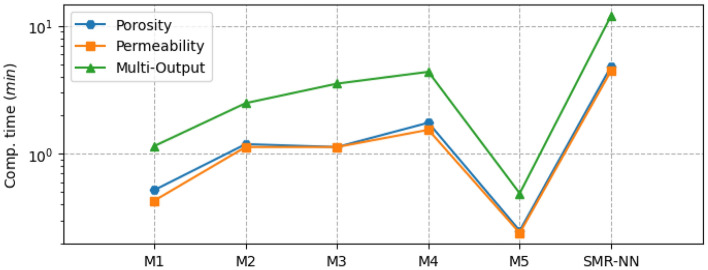
SMR-NN computational time requirement with different target configurations.

**Figure 13:**
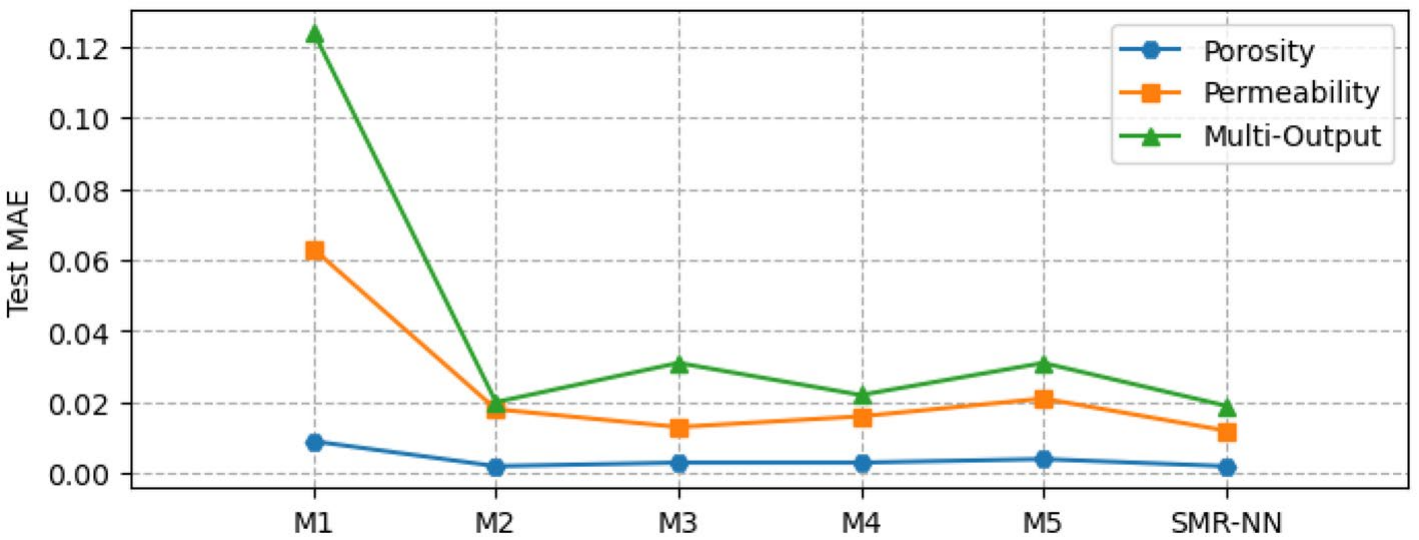
SMR-NN test MAE with different target configurations.

Figure 8 shows the performance of the different selected ML models for single and multi-output configurations, while Fig. 9 shows the corresponding computational time requirement. The R^2^ and computational time of the linear models are significantly low (R^2^ ∼ 0.5). The influence of L1 and L2 regularization is also visible in the implementation of the LR and RG models compared to the MR model in terms of computational time, but there is no significant improvement in model performance. Figure 10 presents the corresponding mean absolute error values for the proposed models tested on unseen data. The test results reflect model performance during training in linear and nonlinear models. Tables 8 and 9 show the overall performance (R^2^ and Test-MAE) and computational time (C. Time) requirements of the proposed stacked models, which were trained using the optimal hyperparameters shown in Table 6. In Fig. 11, the performance of DNN models improves as the model complexity increases. However, this performance improvement increases computational time (Fig. 12). Between models M4 and M5, we observe a decline in computational time with an increase in perceptron dropouts. However, this slightly increases the testing mean absolute error (Fig. 13).

**Table 8:** General performance for the different ML models for both single and multi-output targets.

	MR	LR	RG	RF	GB	SMR-ML	SMR-NN
Single-output (porosity)							
R^2^	0.52	0.52	0.48	0.99	0.99	0.99	0.99
MAE	0.08	0.08	0.09	0.0003	0.0004	0.0012	0.0015
C. Time (min)	0.1	0.01	0.001	2.1	9.6	55.6	4.8
Single-output (permeability [mD])							
R^2^	0.28	0.28	0.25	0.99	0.99	0.99	0.95
MAE	1.13	1.14	1.20	0.0023	0.0003	0.0002	0.012
C. Time (min)	0.4	0.05	0.001	2.3	95.7	229.4	4.5
Multi-output (porosity and permeability [mD])							
R^2^	0.39	0.39	0.39	0.93	0.92	0.93	0.96
Test_MAE	0.61	0.61	0.61	0.11	0.09	0.11	0.02
C. Time (min)	0.3	0.6	0.001	0.2	8.5	10.0	12.1

**Table 9:** General performance for the different DNN models for both single and multi-output targets.

	M1	M2	M3	M4	M5	SMR-ML	SMR-NN
Single-output (porosity)							
R^2^	0.88	0.99	0.97	0.97	0.96	0.99	0.99
MAE	0.009	0.002	0.003	0.003	0.004	0.001	0.002
C. Time (min)	0.5	1.2	1.1	1.7	0.3	55.6	4.8
Single-output (permeability [mD])							
R^2^	0.58	0.90	0.92	0.92	0.90	0.99	0.99
MAE	0.06	0.02	0.014	0.02	0.02	0.0002	0.012
C. Time (min)	0.4	1.1	1.1	1.5	0.3	229.4	4.5
Multi-output (porosity and permeability [mD])							
R^2^	0.77	0.95	0.96	0.96	0.95	0.93	0.96
MAE	0.12	0.02	0.03	0.02	0.03	0.107	0.018
C. Time (min)	1.2	2.5	3.5	4.4	0.5	10.0	12.1

Tables 10, 11, 12, and 13 compare the average prediction of porosity and absolute permeability (from unseen image slices) with single and multi-output stacking approaches values to experimental values. In a single output arrangement, the SMR-NN and the SMR-ML model get promising results with average percentage error values ranging between 0.01–0.12% and 0.01–0.06% for porosity, and 0.22–1.38% and 0.16–15.8% for absolute permeability, respectively. On the other hand, with a multioutput arrangement, the SMR-ML outperforms the SMR-NN model, with average percentage errors for both porosity and absolute permeability ranging between 0.64–1.7% and 1.5–5.93%, respectively.

**Table 10:** SMR-ML model single-output target predictions.

Sample	Experiments		Prediction		Av. % error
	Porosity	Permeability	Porosity	Permeability (mD)	
BB	0.257	11.30	0.257	12.21	4.07
SD	0.158	278.85	0.158	279.30	0.10
ALB	0.208	10.23	0.208	8.61	7.91
TC	0.256	336.94	0.256	337.60	0.13

**Table 11:** SMR-NN model single-output target predictions.

Sample	Experiments		Prediction		Av. % error
	Porosity	Permeability	Porosity	Permeability (mD)	
BB	0.257	11.3	0.257	11.44	0.64
SD	0.158	278.85	0.158	278.13	0.16
ALB	0.208	10.23	0.208	10.09	0.69
TC	0.256	336.94	0.256	336.20	0.17

**Table 12:** SMR-ML model multi-output target predictions.

Sample	Experiments		Prediction		Av. % error
	Porosity	Permeability	Porosity	Permeability (mD)	
BB	0.257	11.30	0.257	11.01	1.29
SD	0.158	278.85	0.157	283.95	1.23
ALB	0.208	10.23	0.208	10.57	1.70
TC	0.256	336.94	0.257	333.96	0.64

**Table 13:** SMR-NN model multi-output target predictions.

Sample	Experiments		Prediction		Av. % error
	Porosity	Permeability	Porosity	Permeability (mD)	
BB	0.257	11.3	0.237	11.84	1.50
SD	0.158	278.85	0.150	280.29	2.27
ALB	0.208	10.23	0.216	11.15	5.93
TC	0.256	336.94	0.249	318.29	4.13

**Figure 14:**
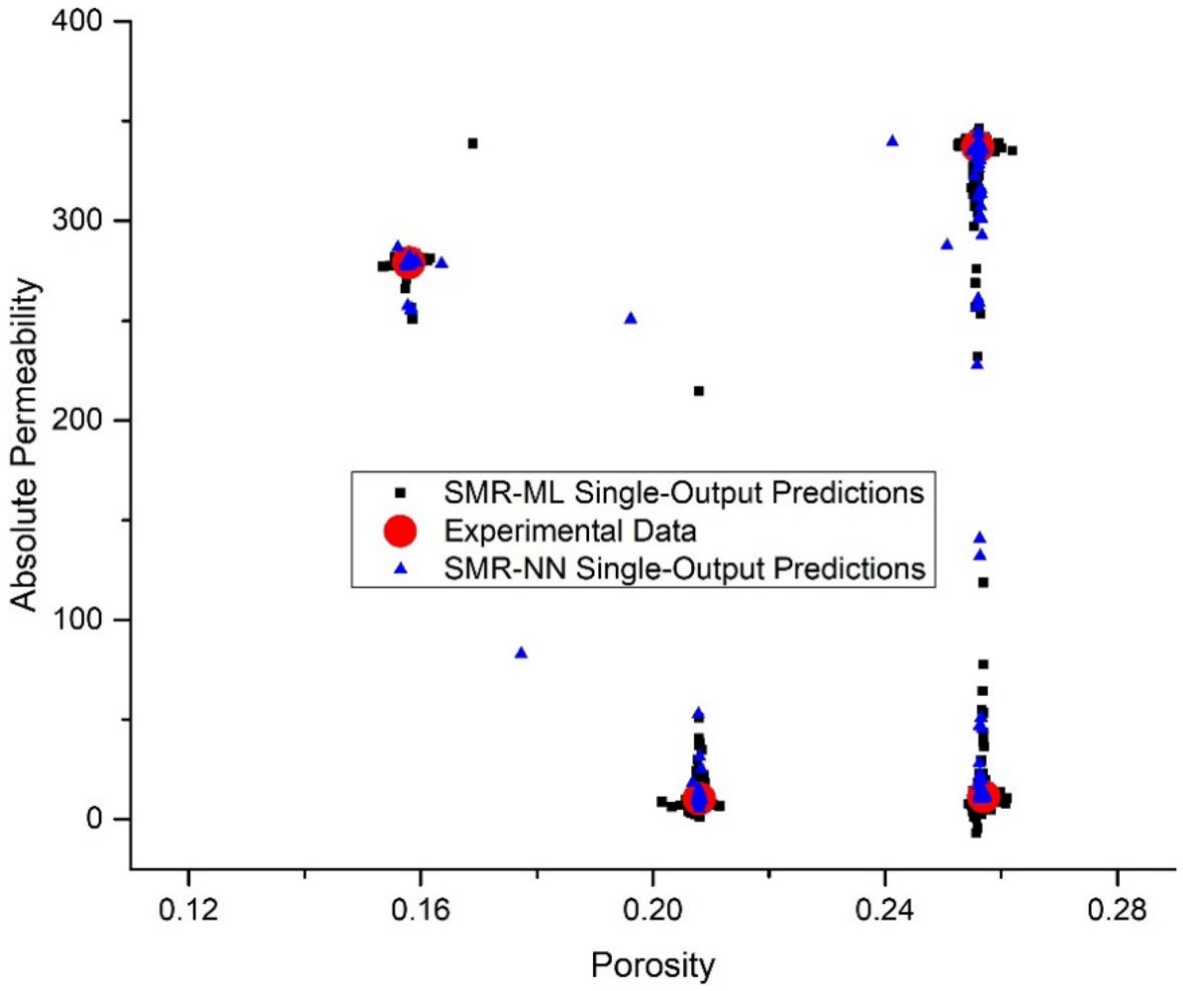
Permeability–porosity cluster plot demonstrating the robust prediction capabilities of the single output SMR-ML and SMR-NN models.

**Figure 15:**
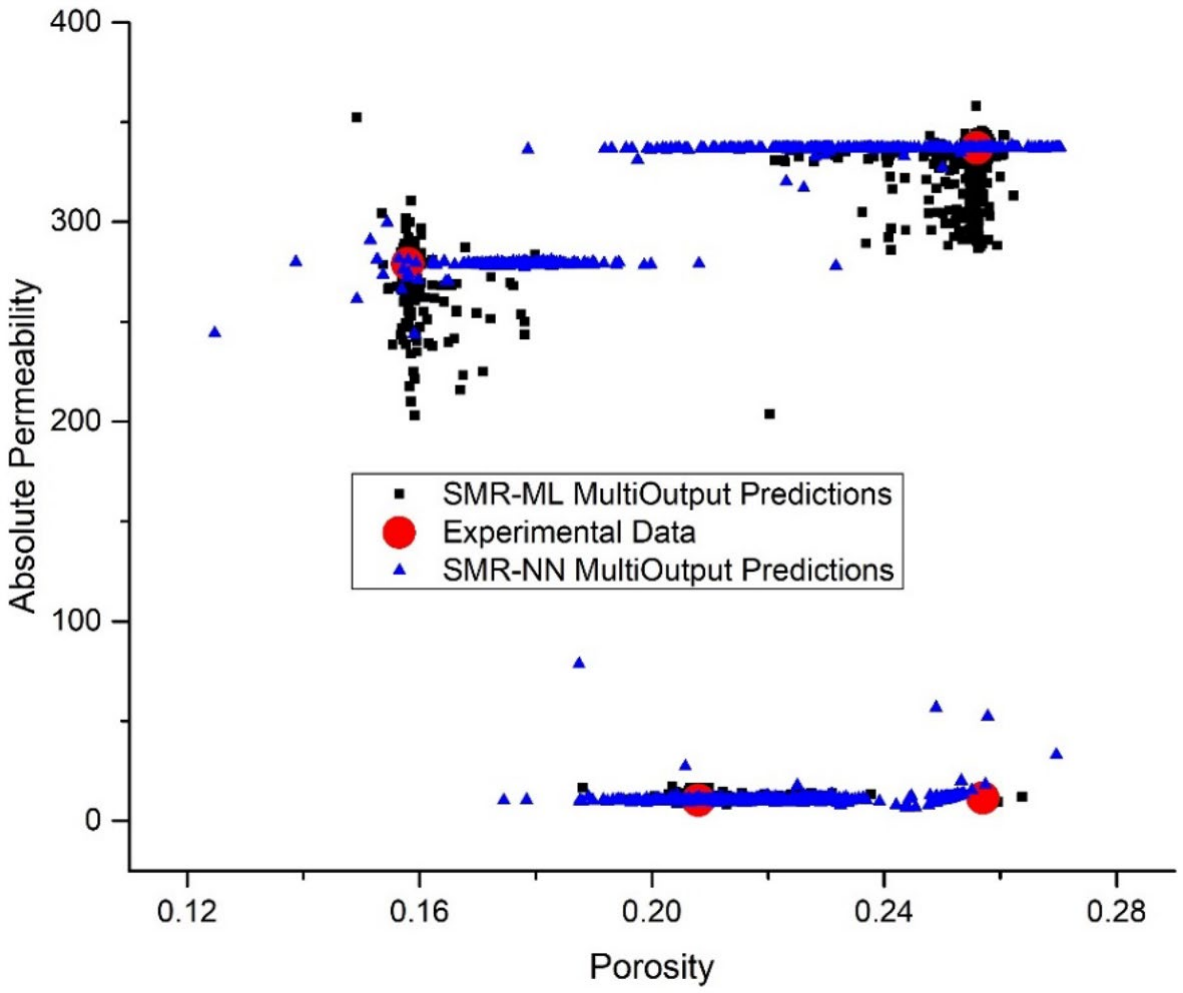
Permeability–porosity cluster plot demonstrating the robust prediction capabilities of the multioutput SMR-ML and SMR-NN models.

Figures 14 and 15 present a permeability–porosity cluster plot demonstrating the robust prediction capabilities of the SMR-ML and SMR-NN models for single and multioutput arrangements, respectively. The plot showcases the accuracy of the models in predicting permeability and porosity values from the testing (unseen) dataset while highlighting the tight clustering of the predicted values to the true values, indicating their consistency and reliability.

## Discussions

In machine learning models, results show a strong nonlinear relationship between the input features and all the targets. Regarding computational resources, nonlinear models require higher computational time to train than linear models. Interestingly, when we focus on both linear and nonlinear ML models, we see that linear models' predictive capability is relatively limited for single and multi-output considerations. We also observe that adding regularization hyperparameters to the MR model to form RG and LR decreases the computational training requirement of the model (Fig. 8). However, this presents no significant improvement in model performance, especially the RG model, with a decline registered (Fig. 7).

On the other hand, the robustness achieved in both RF and GB due to the accumulation of performances from several estimators enables them to capture the nonlinearities in the dataset. Regarding stacking, the approach yields better performance and predictive accuracy. However, the tradeoff is that this approach requires more computational time to train than the original linear model (MR) and the proposed individual models. Results show that the generalizability error of individual deep neural network (DNN) models can vary considerably during training. Therefore, quantifying the model's complexity is essential to guarantee precision. By stacking multiple individual DNN models, we obtain a more robust model that improves generalizability and predictive power. This method is also more efficient computationally than stacking machine learning models. However, we identify that even with poor-performing weak learners, the SMR-ML model outperforms the SMR-NN regarding predictive accuracy, particularly in the multioutput arrangement. This improved performance of SMR-ML over SMR-NN may be attributed to the bias-variance tradeoff, in which DNNs are likely to present higher variances, which can lead to more diverse predictions compared to machine learning models.

Regarding the output size, both SMR-ML and SMR-NN models could accurately predict porosity values in a single output arrangement. However, SMR-ML struggled to accurately capture the wide range of permeability values, as seen in Fig. 14. This could be due to the strong nonlinear relationship between the inputs and the permeability values. On the other hand, SMR-NN could capture a wide range of permeability values but at the expense of porosity values. In the multioutput arrangement, SMR-ML could predict porosity values accurately, but it tended to under-predict absolute permeability, particularly at high values. Overall, the results suggest that SMR-NN may be a better choice when predicting permeability values in this dataset due to its ability to capture the nonlinear relationships in the data. However, SMR-ML remains a good option for predicting porosity values.

The results show that the meta-learner learned using trained, weaker learners can improve model performance and generalizability. We also observe that stacking independent models takes prohibitive time for training. Considering our approach is based on 2D slices of very complex carbonate rock micro-CT images, these results encourage the adoption of stacked ensemble learning for the petrophysical data determination of core plugs.

Our primary goal in this study is to show that stacked ensemble machine learning models outperform traditional machine learning models for predicting carbonate rock formations' porosity and absolute permeability. However, we identified some limitations associated with this study. First, like any machine learning implementation, the accuracy of the prediction models heavily depends on the quantity and quality of the input data. Factors such as the normalization techniques and data partitioning strategy can also impact the model's performance. In this study, for instance, we combined data from multiple core samples and randomly selected them for training and testing, which may lead to overestimating or underestimating the model's performance. Second, the stacked ensemble machine learning approach can be computationally expensive and time-consuming, posing challenges for specific applications with limited computational resources. Therefore, considering the computational requirements and time constraints when applying this approach in practical scenarios is essential. Third, we acknowledge that the heterogeneity of carbonate reservoirs can be substantial; therefore, model prediction might not accurately reflect the whole reservoir's properties. Increasing the dataset's number of 3D core image samples from various spatial locations of the reservoir could remedy this issue. In subsequent works, we plan to use deep convolutional neural networks to predict absolute permeability and porosity using actual carbonate image data. In addition, we plan to investigate the impact of transfer learning, model size, and dataset size on performance and prediction accuracy.

## Conclusion

The present study highlights the limitations and challenges associated with predicting petrophysical properties from 2D images for reservoir characterization and proposes stacked ensemble machine learning as a workflow to increase the predictive accuracy of 2-D image analysis. We showed that combining stacked ensemble machine learning models and well-established image analysis techniques (image pore properties or RegionProps) can enhance traditional machine learning methods' predictive accuracy and effectiveness. Perhaps it is worth highlighting that the proposed stacked ensemble machine learning is applied in the context of carbonate rock formations, which pose challenges due to their inherent heterogeneity and complex pore structures and where the applications of statistical and machine learning techniques to predict porosity and permeability are limited.

In this paper, we developed a workflow and presented the capabilities of various ML models, including DNNs, to predict carbonate rocks' absolute permeability and porosity. We utilized a large dataset of pore features extracted from 2D slices of 3D micro-CT images of four complex carbonate core plugs. To minimize model variances and mitigate overfitting, we used a novel ML approach (stacking) that integrates several ML and DL models to predict porosity and absolute permeability. We compared ML-based, DNN-based models and stacking methods regarding performance and computational time requirements. Obtained results show that both SMR-ML and SMR-NN can outperform the individual proposed models regarding predictive accuracy. However, results also show that the computational time of stacked models is generally higher than individual models. Therefore, the choice between stacked ensemble and single models should be made based on a tradeoff between prediction accuracy and computational efficiency.

Furthermore, we found that stacking workflow improves model generalizability. We also found that the DNNs perform slightly better than the individual ML models. This means the linear models perform and generalize less than the nonlinear ones, requiring higher computational time. Finally, we show that stacked models can predict permeability and porosity with average errors of 1.2% for SMR-ML and 3.5% for SMR-NN models. This study provides a workflow for predicting the petrophysical properties of complex rock samples based on micro-CT images. With a trained ML model, predicting target properties can take a few seconds compared to the time and cost-consuming numerical simulations and experiments.

## Data Availability

Data and codes accessible vias: Stacking-Ensemble: https://github.com/kalx-cyber/Stacking-Ensemble.
